# The Question Answered

**Published:** 1881-06

**Authors:** C. Carleton Smith

**Affiliations:** Philadelphia


### THE QUESTION ANSWERED.

C. CARLETON SMITH, M.D., PHILADELPHIA.

THE following question was put, to the homœopathic profession at large, by Dr. T. P. Wilson, in the *Medical Advance*, Dec., 1880, p. 283.

"What can be done for the instant or rapid relief of our patients suffering acute pain? Will those who do not use narcotics please answer and illustrate?"

As I do not, and never have, used narcotics in the course of an active practice extending over a period of nineteen years, I feel that I, for one at least, can with propriety answer this question.

When called to a case in which I find very acute suffering from pain, no matter what the nature of these pains may be, provided they are cases requiring purely medicinal treatment and not mechanical, I calmly and deliberately sit down and examine the case before me with the greatest minuteness and care, never heeding that oft-repeated clamor of the attendants, so often heard in the sick-room that "something must be done or the patient will die." The greater the suffering the more particular am I in my examination, in order to get at the proper homœopathic specific in the most direct way. One of the greatest blunders that a homœopathic practitioner can be guilty of in such cases as these, is to allow himself to be dictated to in the sick-room, and thereby make hap-hazard prescriptions for the purpose simply of deluding those present into the belief that he is really doing something for his patient, thereby losing many precious moments, prolonging the patients sufferings, and spoiling his case into the bargain. I have no hesitation in asserting that a physician who, upon being ushered into the pres ence of such cases as these now under consideration, simply glances at the patient whom he had never, up to this moment, heard of before, much less seen, pulls out his pocket case and gravely asks for a half-glass of water and a spoon with which to prescribe, does not by any means show his smartness, but on the contrary he exhibits his ignorance and is unworthy, by his own showing, of the name of physician. Nor on the other hand does the physician who on the spur of the moment applies a sinapism or calls for turpentine with which to anoint his patient, or chloroform to render him unconscious, exhibit very great learning, but on the contrary he betrays both his weakness and his total unfitness for the work before him.

When people undertake to push me, in the sick-room, I plainly but politely tell them that I must know exactly what the patient requires in order to relieve him of his great distress, before I prescribe, and if I am not allowed to make the proper examination with this end in view, which is a duty I owe both to myself and patient, I must respectfully refuse to have any thing further to do with the case.

From what I have already written, you will perceive that I prefer, and do rely upon, the strictly homœopathic remedy in each and every case of emergency, and wholly abjure all palliatives with the occasional exception, sometimes, of hot or cold water. I never keep narcotics in my office, nor do I carry them in my pocket case; and I can ask no better satisfactien than I have enjoyed in the practice of my profession, in relieving acute pain, no matter how sudden its onset, quickly and permanently.

Illustrations.—I was called to visit a gentleman eight years ago, suffering from the passage of a urinary calculus down the right ureter. The agony which he endured, shown by his writhings and the cold beaded sweat upon his pallid face, I shall never forget. He had several attacks of a similar nature previous to this, within a period of two years, each on the *right side.* I gave him, after careful study, a single dose of lyc. 30, in water. Almost instantly after the drug was swallowed the patient turned over on his left side with his face to the wall and fell into a sound, peaceful sleep, so sound and so quiet that his good wife thought he was dead. After he reposed in this way for one hour he got up, passed his urine freely, which before was voided in drops and loaded with red sand; and never, from that day to this, has this sufferer had another attack. Are there any palliatives which can excel this treatment?

Another case was that of the passage of gall stones in the person of a Miss of eighteen years. I found her in bed screaming fearfully, tearing her hair, and rolled up in the shape of a ball like a hedge-hog, so great was her agony. China was the remedy here, which she received in the 20 M. potency, repeated a few times in water; relief soon came, and next day when I called, the young lady herself opened the front door and ushered me into the house with her face wreathed with smiles. The symptoms leading me to the use of this drug were, first, icterus, and added to this there was *extreme depression* and *want of breath* with desire to be fanned, pain in region of liver worse from least touch.

Again, I was called one evening to see a little five year old girl afflicted with Parotitis of the most aggravated type. Both sides were involved, and the pain was so exquisitely severe that the sufferer sat up in bed screaming constantly, keeping every one at a distance for fear of being touched. She could not bear the band of her night dress to come in contact with any portion of her neck, and would not allow me to even point my finger at the swollen glands without screaming and becoming almost convulsed with pain and terror combined.

Now what palliative could be placed upon a surface like that? There is but one answer to this question, *Not any.* Lachesis

2 C., in water gave immediate relief, which was followed by sleep, and the following day I was not only allowed to touch the neck but also to knead it with my fingers as though it had been so much dough.

Some years since I stood by the bedside of a young woman to whom Dr. Hering had been summoned in great haste. The nature of the case was puerperal convulsions. The tongue being protruded between the teeth and the jaws locked, the face purple and the breathing stertorous, the doctor remarked that no remedy could be administered by the mouth. What did he do? resort to some palliative? No: the grand old man took from his pocket-case a tiny vial containing a few pellets of opium 2 C. which he held to one of the nostrils of the patient, while he closed the other with his finger. After a few inhalations the spasms ceased instantly and the storm was calmed.

Would morphia or any palliative have acted better than the remedy whose pathogenesis was similar to the characteristics of the case?

Adjuvants in the shape of narcotics and local appliances of a medicinal character do not belong to the homœopathic law of cure as it was handed down to us by the master, yet we find that they invariably follow in the wake of that class of so-called doctors of homœopathic medicine who are continually being graduated from institutions ostensibly Hahnemannian, but within whose walls, notwithstanding, Hahnemann's "Organon" is *never studied*, or even commented upon.

If a man turns his face away from the light, he is in darkness; and of such are those who willfully turn their backs upon the law of the similars.

